# Advanced Stage at Presentation Remains a Major Factor Contributing to Breast Cancer Survival Disparity between Public and Private Hospitals in a Middle-Income Country

**DOI:** 10.3390/ijerph14040427

**Published:** 2017-04-16

**Authors:** Yek-Ching Kong, Nirmala Bhoo-Pathy, Shridevi Subramaniam, Nanthini Bhoo-Pathy, Nur Aishah Taib, Suniza Jamaris, Kiran Kaur, Mee-Hoong See, Gwo-Fuang Ho, Cheng-Har Yip

**Affiliations:** 1 National Clinical Research Centre, Ministry of Health, Kuala Lumpur 50586, Malaysia; stella_kyc@hotmail.com (Y.-C.K.); shridevi@crc.gov.my (S.S.); 2 Department of Social and Preventive Medicine, University of Malaya, Kuala Lumpur 50603, Malaysia; ovenjjay@gmail.com (N.B.-P.); nandy1410@gmail.com (N.B.-P.); 3 Department of Surgery, Faculty of Medicine, University of Malaya, Kuala Lumpur 59100, Malaysia; nuraish@gmail.com (N.A.T.); ahshun82@gmail.com (S.J.); kkaur79@yahoo.com (K.K.); smhoong76@gmail.com (M.-H.S.); 4 Department of Clinical Oncology, Faculty of Medicine, University of Malaya, Kuala Lumpur 59100, Malaysia; gwofuang@gmail.com; 5 Subang Jaya Medical Centre, Subang Jaya, Selangor 47500, Malaysia

**Keywords:** breast cancer, overall survival, public vs. private hospital

## Abstract

*Background:* Survival disparities in cancer are known to occur between public and private hospitals. We compared breast cancer presentation, treatment and survival between a public academic hospital and a private hospital in a middle-income country. *Methods:* The demographics, clinical characteristics, treatment and overall survival (OS) of 2767 patients with invasive breast carcinoma diagnosed between 2001 and 2011 in the public hospital were compared with 1199 patients from the private hospital. *Results:* Compared to patients in the private hospital, patients from the public hospital were older at presentation, and had more advanced cancer stages. They were also more likely to receive mastectomy and chemotherapy but less radiotherapy. The five-year OS in public patients was significantly lower than in private patients (71.6% vs. 86.8%). This difference was largely attributed to discrepancies in stage at diagnosis and, although to a much smaller extent, to demographic differences and treatment disparities. Even following adjustment for these factors, patients in the public hospital remained at increased risk of mortality compared to their counterparts in the private hospital (Hazard Ratio: 1.59; 95% Confidence Interval: 1.36–1.85). *Conclusion:* Late stage at diagnosis appears to be a major contributing factor explaining the breast cancer survival disparity between public and private patients in this middle-income setting.

## 1. Introduction

The two most important determinants of cancer survival are early detection and optimal access to treatment. Discrepancies in breast cancer survival have been known to occur between developing and developed countries, due to late presentation and poor access to care in developing countries. The Cancer survival in five continents: a worldwide population-based study (CONCORD-2 study) analyzed the data of 25,676,887 patients from 279 population-based registries in 67 countries and found that the five-year survival of breast cancer patients diagnosed between 2005 and 2009 was 80% and higher in high-income countries compared to 68% in Malaysia, 60% in India, and 53% in South Africa, which are low- and-middle income countries [1]. A study on cancer incidence and mortality in the 27 member states of the European Union, where attempts have been made to standardize health care, showed that better outcomes were associated with a higher health expenditure [2].

Survival discrepancies within the same high-income country are also known to occur. For example, in the United States (U.S.), survival is consistently worse in American blacks compared to American whites. A study using the SEER-Medicare database showed that the absolute difference in the breast cancer five-year survival rate was 12.9% between blacks and whites when matched for demographic characteristics. This difference, however, decreased to 4.4% after matching on cancer presentation characteristics and to 3.6% when matched for treatment details, concluding that the difference in breast cancer survival between blacks and whites in the U.S. appear primarily to be related to presentation characteristics at diagnosis rather than treatment differences [3]. Poorer survival in disadvantaged patients is even more pronounced in cancers with better prognoses, such as female breast cancer [4].

A hospital-based study in Malaysia found that breast cancer survival is poorer in Malay patients compared to Chinese and Indian patients, independent of stage at diagnosis, tumor characteristics and treatment [5]. Comparison of breast cancer outcomes between Malaysia, an upper middle-income country, and Singapore, a high-income country, showed that the five-year survival rate was 69% in Malaysia compared to 80% in Singapore. Even after adjusting for prognostic factors, Malaysian patients were at a 67% increased risk of death compared to Singaporeans [6]. 

Malaysia is one of the few countries in South-East Asia that has universal health care (UHC) provided through the public service, but it also has a separate private health care system which is paid by private insurance or out of pocket (OOP). In 2014, Malaysia spent 4.2% of its Gross Domestic Product (GDP) on health care, which is 456 USD per capita. It has, however, been revealed that the OOP health expenditure in Malaysia is high. More than 30% of health care financing in the country is OOP, suggesting that there are challenges in the UHC provided by the Malaysian government [7]. It is likely that overcrowded public hospitals along with long waiting times [8] have pushed Malaysian patients to pay OOP in order to seek treatment in private hospitals. 

University of Malaya is the oldest public university in Malaysia. It has its own teaching hospital, known as the University Malaya Medical Centre (UMMC) that was opened in 1968, where clinicians are appointed as academic staff. The clinicians, however, tend to leave after an average of five years’ service for the more lucrative private sector due to high workload and low salaries in the public sector. In 2000, in an attempt to avoid this “brain drain” in the university, a private wing was started (University Malaya Specialist Centre (UMSC)) where clinicians from UMMC are able to practice after office hours to increase their income. Patients seeking treatment in UMMC receive heavily subsidized treatment and, if they are government servants, treatment is provided free of charge. UMSC, on the other hand, functions as a fully privatized hospital and patients either pay out of pocket or are covered by third party payers. Patients with breast cancer are managed overall by a single consultant breast surgeon in both the private and public wing of the University of Malaya. This therefore provides an excellent setting for the comparison of presentation, treatment and outcome following breast cancer between the public and private settings in Malaysia, which is the objective of this study. 

## 2. Materials and Methods 

After the exclusion of in situ cases, 2767 breast cancer patients treated in the University Malaya Medical Centre, a public tertiary hospital, were compared with 1199 patients treated in the University Malaya Specialist Centre, which is the private wing of the same hospital, over the same time period; January 2001 to December 2011. Data for the present study was retrieved from the University Malaya Breast Cancer Registry, which is a prospective hospital-based breast cancer registry including patients from both UMMC and UMSC. Further details of the registry have been previously described [9]. The University Malaya Breast Cancer Registry is approved by the institution’s Medical Ethics Committee (Ref. No. 733.23). Consecutive new cases of breast cancer are entered into the registry, and it is considered complete because it is under the same consultant breast surgeon in both the private and public wing of the University Malaya. 

Demographics and clinical characteristics of breast cancer patients from UMMC and UMSC were compared using Chi-square test and logistic regression. In addition, multivariable logistic regression analysis with receipt of standard treatment as the outcome (yes, no) was also performed to compare patients from UMMC to UMSC. Standard treatment was defined using the National Clinical Practice Guideline (CPG) for Breast Cancer [10], i.e., surgery for non-metastatic patients; chemotherapy if node positive, Grade 3, estrogen receptor (ER) or progesterone receptive (PR) negative, human epidermal growth factor positive (HER2), or a tumor size of more than 2 cm; radiotherapy after breast-conserving surgery; and hormone therapy for patients with ER positive tumors. The only factors that were significantly associated with the respective adjuvant treatment were included and adjusted for in the respective models. 

Data on mortality were verified through linkage with the National Registration Department in Malaysia. In this hospital-based cancer registry, information on cause of death or cancer recurrence was not available for the majority of patients. Less than 50% of deaths in Malaysia are medically certified, as deaths at home are certified by laymen, usually the police, and may not be accurate. Hence, overall survival rather than breast cancer-specific survival is the outcome used in this study. Information on whether the patient is alive or dead is accurate given that reporting of death is mandatory in Malaysia. Follow-up time was calculated from the date of diagnosis of breast cancer to the date of death or censored at the end of the follow-up (March 2016). 

Kaplan-Meier analysis and log rank test were used to compare overall survival. A crude Cox regression model was built to estimate the relative risk of all-cause mortality among breast cancer patients between the two settings (public vs. private hospital). Stepwise adjustment of the initial model for patient characteristics (age, ethnicity and year of diagnosis), cancer stage at diagnosis (tumor size, number of regional nodes positive and distant metastasis) and details of cancer therapy (locoregional therapy, chemotherapy, and hormone therapy) was undertaken. The changes in the hazard ratio (HR) for type of setting between the initial and stepwise models were used to gauge the extent to which patients’ characteristics, cancer stage, and cancer therapy, respectively explained the variation in all-cause mortality among the breast cancer patients treated in the public and private settings. 

In this study, missing-indicator method was used to deal with missing data [11]. For a given variable, missing observations were assigned to an additional dummy category and included in the analytical model. This avoids patients with missing data being excluded from the analysis, thus maintaining the statistical power of the study. 

All analyses were performed using SPSS version 22 (IBM Corp, Armonk, NY, USA) A *p*-value of <0.05, and 95% confidence interval (CI) for odds ratio (OR) or HR that does not include 1.0, were considered statistically significant.

## 3. Results

### 3.1. Demographics and Clinical Characteristics

Invasive breast cancer patients from the public hospital presented at an older age (median age of 53 years vs. 50 years, respectively) and with larger tumors (median size of 3 cm vs. 2.5 cm, respectively) when compared to patients from the private hospital (Table 1). Patients from the public hospital were significantly less likely to be Chinese. They were also more likely to be diagnosed at more advanced cancer stages than their counterparts managed in the private practice; Adjusted Odds Ratio (adjOR) for presentation with stage III disease: 1.71; 95% CI 1.39–2.11, and adjOR of 3.30; 95% CI 2.38–4.58 for de novo stage IV breast cancer.

### 3.2. Treatment

Among breast cancer patients with non-metastatic cancer that underwent surgery, a higher proportion in the public hospital received mastectomy (76.4% vs. 70.6%, respectively; *p*-value < 0.001) while more private patients underwent breast conserving surgery (BCS) (29.0% vs. 23.6%, respectively; *p*-value < 0.001) (Table 2). A significantly higher percentage of public patients were found to have received radiotherapy after breast conserving surgery and mastectomy compared to patients in the private hospital (87.2% vs. 79.4%, respectively after breast conserving surgery and 54.4% vs. 48.2%, respectively after mastectomy; *p*-value < 0.001 for both). However, after adjustment for demographics, tumor and other treatment characteristics, non-metastatic breast cancer patients from the public hospital were found to be less likely to receive radiotherapy compared to the private patients (adjOR 0.83; 95% CI 0.70–0.99). A larger majority of public patients received chemotherapy for stage I–III disease (63.8% vs. 58.0%, respectively; *p*-value < 0.001). Significant differences in type of chemotherapy administration were observed between both institutions, whereby patients from the public hospital were more likely to receive second generation anthracyline-based regimes, while patients from the private hospital were substantially more likely to receive third generation taxane-based chemotherapy regimes.

### 3.3. Survival

The five-year overall survival for patients in the public hospital was substantially lower compared to that of the private hospital patients (71.6% vs. 86.8%, respectively; log rank test *p* < 0.001). This was also seen when comparing the ten-year overall survival rates between patients from the public and private hospitals (52.3% vs. 76.6%, respectively; log rank test *p* < 0.001) (Table 3). When stratified by stage, the five-year and ten-year overall survival estimates of patients from the public hospital remained significantly lower compared to their counterparts in the private center (log rank test; *p* = 0.036 for stage I, *p* < 0.001 for stage II and III, *p* = 0.005 for stage IV). 

Mortality risk was more than two-fold higher among the patients in the public hospital compared to the private hospital patients (crude hazard ratio 2.22; 95% CI 1.94–2.55). After adjusting for demographics (age, ethnicity and year of diagnosis), clinical characteristics (tumor size, number of regional nodes) and treatment (surgery, radiotherapy, chemotherapy and hormone therapy) in a stepwise multivariable Cox regression analysis, the risk of death among UMMC patients was attenuated (adjusted hazard ratio (adjHR) 1.59; 95% CI 1.36–1.85) (Table 4). Approximately 20% of the discrepancies in risk of mortality between patients in the two hospitals were attributed to stage at diagnosis, while only 7% of the survival differences appeared to be explained by patient demographics, whereas differences in treatment received explained 4% of the survival disparity. 

## 4. Discussion

Our study suggests that late stage at diagnosis is a major contributing factor explaining the breast cancer survival disparity between public and private patients in this middle-income setting. It should, however, be noted that even after adjusting for differences in demographics, clinical characteristics and treatment between the two settings, breast cancer patients in the public sector remained at increased risk of mortality compared to their counterparts in the private practice.

There is a discrepancy in access to optimal care between the rich and the poor even in high-income countries, as seen in a study on 4675 breast cancer patients in New Jersey, USA, who were diagnosed between 1985 and 1987. In this study, the survival of patients with local and regional disease was significantly worse among uninsured patients and those with Medicaid coverage compared with those who were privately insured. This suggested that women without private health insurance would benefit from improved access to screening and optimal therapy [12]. 

In the present study, patients from the public sector were more likely to present at an older age, and with larger tumors and later stages compared to those in the private sector. This was also reported in a study in the U.S., where uninsured patients presented with larger tumors and higher proportion of node positivity compared to women with private insurance [13]. 

In a study by Coburn et al., women with private insurance were more likely to have breast conserving surgery and breast reconstruction after mastectomy compared to the uninsured women. Furthermore, uninsured women were also more likely not to have any surgical treatment [13]. This was also seen in the current study, where a significantly higher percentage of private patients with non-metastatic tumors underwent breast conserving surgery. while more public patients underwent mastectomy. However, this could be because public patients presented with a larger tumor size (3 cm) compared with private patients, who had a median size of 2.5 cm, and this would have made breast conservation more difficult in the public patients.

When type of chemotherapy use in the two settings was studied, public patients were found to be less likely to receive third generation regimes, but more likely to receive second generation regimes. Third generation regimes include the addition of a taxane to anthracyclines, whereas second generation regimes contain only anthracyclines. Notably, clinical trials have demonstrated an improvement in disease-free and overall survival with use of third generation regimes in the adjuvant setting [14]. The reason for the difference in type of chemotherapy regime between the two settings was that generic taxanes were not available during the study period, and taxane-based chemotherapy was not subsidized in the University Malaya Medical Centre, requiring patients to pay OOP in order to obtain it. Patients who were unable to afford taxanes received anthracycline-based chemotherapy alone.

While more public patients appeared to receive radiotherapy compared to private patients, after adjustment for demographic, tumor and other treatment characteristics, public patients were found be significantly less likely to receive radiotherapy. Although radiotherapy is offered at highly subsidized rates in the public hospital, the lack of radiotherapy use in the public setting may be explained by psychosocial factors including fear of radiotherapy, as well as due to logistic issues given that patients receiving radiotherapy are required to commute to the hospital more often, incurring extra expenses and absence from work/social obligations. Patients in the private sector, on the other hand, may be better able to afford second opinions from other oncologists/physicians to allay their fears, and are more likely to be economically stable and therefore able to incur the additional costs associated with radiotherapy for transportation, childcare, medical leave, etc. Since the private and public centers are geographically next to each other, and the majority of the patients are from the surrounding districts, the distance from home to hospital is similar in both groups. 

Hormone therapy use was similar in both groups, which is likely to be because tamoxifen is a cheap treatment available in both public and private settings. We did not analyze the use of trastuzumab because its use was very low during the study period as most patients were unable to afford it, including the private patients. A nationwide study in Malaysia previously showed that only 15% of eligible patients in the country received trastuzumab [15]. 

The five- and ten-year survival rates in the public setting was substantially lower compared to the private setting, with a more than two-fold higher mortality risk in the public patients (crude hazard ratio: 2.22; 95% CI 1.94–2.55). After adjusting for demographic, tumor and treatment differences, the mortality risk was still 59% higher in the public patients. This discrepancy is much higher than a similar study conducted in New Zealand, where the crude hazard ratio of 1.95 was attenuated to a 14% higher risk of mortality in the public sector after adjusting for ethnicity, stage at diagnosis and type of locoregional treatment [16]. The increased risk in mortality, independent of stage and treatment, could be attributed to disparities in socioeconomic status and differences in psychosocial characteristics between the patients in public and private settings, which may together affect the health-seeking behavior, access to expensive treatment, adherence to treatment, lifestyle after cancer and quality of life of breast cancer patients. An ongoing prospective breast cancer cohort study in our setting is expected to shed some light in this area [17]. 

Survival from breast cancer is mainly influenced by early diagnosis and access to treatment, with tumor biology playing a more minor role. When we look at each of these three factors, the survival disparity observed in the present study appear to be largely attributed to discrepancies in stage at diagnosis, and only to a much smaller extent by demographic differences and treatment disparities. In the study in New Zealand, ethnicity, stage at diagnosis and type of locoregional therapy were the three key contributors to survival disparities between public and private patients. However, the relative contributions of each factor were not explored in the study. The SEER-Medicare database however showed that the difference in the five-year survival rate between blacks and whites appear primarily to be related to presentation characteristics at diagnosis rather than treatment differences, as was demonstrated in the current study [3]. 

Although access to treatment is another important factor for improved survival, there was only a small discrepancy in survival based on treatment, although the private patients had access to third generation regimes. Third generation regimes such as sequential adjuvant chemotherapy with FEC (5-fluorouracil, epirubicin and cyclophosphamide) followed by docetaxel significantly improved disease-free and overall survival in node-positive breast cancer patients compared with second generation regime (FEC alone). However, the absolute difference in survival was very small [18]. Discrepancy in access to third generation regimes indeed did not explain the survival disparities between public and private patients in the current study. Stage at diagnosis varies the most between the private and public patients, and disparities in cancer treatment only contributed to a small difference in overall survival between the public and private patients, meaning that provision of cancer care by a multidisciplinary team in a public hospital may be just as good as in a private hospital. The oncology unit and the breast unit in the University Malaya Medical Centre run a combined clinic that follows the national clinical practice guidelines for the management of breast cancer. Unfortunately, this multidisciplinary model is often not present in most parts of the country due to a lack of oncologists and breast surgeons. 

Based on our study, the major focus to improve survival and reduce disparities between public and private patients would be to advocate for early detection in women in the lower socioeconomic group and uninsured patients. With a median tumor size of 3 cm, mammographic screening is not likely to play a major role in early detection, even if it is feasible in a low- or middle-income country (LMIC). With a large palpable tumor, downstaging by breast self awareness (including breast self examination) and clinical breast examination are likely to play a more important role in early detection. Moreover, the impact of screening mammography has not been studied in LMICs. Hence, more effort and resources must be put into a public education program to improve breast health literacy [19].

Several non-governmental organizations in Malaysia are involved in early detection programs for breast cancer, while some advocacy groups are fighting for better access to expensive targeted therapy for breast cancer. If there is only a limited budget for breast cancer control then, based on the results of this study, a fair proportion of the budget should be for public health programs to detect breast cancer as early as possible. 

## 5. Limitations

The data on stage, clinical characteristic and treatment was derived from the University Malaya Database, which is a prospectively collected database. However, data on timeliness of treatment, that is, patient or systems delays, were unfortunately not collected. Data on socioeconomic status was not collected, and we merely assumed that breast cancer patients who were seen in the private setting were better educated, came from higher income groups, and had private insurance. We are also limited by the lack of data on smoking, co-morbidities, access to social support, lifestyle, and body mass index between the two groups. All of these factors could have contributed to the 59% higher risk of mortality in the public patients. 

Data on cause of death is not available. However, as the majority of patients are young, with a median age of 52, competing risk of death from other causes are likely to be minimal. As such, our main inference is likely to remain the same even with the use of overall survival estimates. 

In the stepwise analysis on treatment differences between the two centers, HER2 status was not included because patients were not treated differently due to HER2 status as trastuzumab treatment was not available in both settings. 

This study was carried out in only two centers, both of which have excellent facilities, and hence may not reflect the situation in the rest of the country. Notably, a population-based registry study in Malaysia had shown a five-year overall survival of only 49% among breast cancer patients diagnosed between 2000 and 2005 [20]. 

## 6. Conclusions

There is a large discrepancy in breast cancer survival between the public and private hospital setting; which was attenuated to a 59% higher risk of mortality in the public sector following adjustment for stage, demographics and treatment. From this study, the main contributor to the survival discrepancy between public and private patients appears to be late stage at diagnosis. Policies on breast cancer control in the country therefore need to focus more on efforts to improve early detection in order to reduce the survival disparity between private and public hospitals, which must be closely tied with the provision of affordable cancer care. Apart from the above factors, further research is needed to determine other possible contributors, such as timeliness of treatment, co-morbidities, body mass index, and lifestyle factors after a diagnosis of breast cancer, which may explain the excess mortality risk in the public setting compared to the private setting.

## Figures and Tables

**Table 1:** Distribution of patient demographics and clinical characteristics by hospital type.

	Overall (n = 3966) n, %	UMMC (n = 2767) n, %	UMSC (n = 1199) n, %	Adjusted OR (95% CI) ^a^
**Age of diagnosis (Years)**				
Median (range)	52 (23–95)	53 (23–95)	50 (23–92)	
<40	477 (12.0%)	340 (12.3%)	137 (11.4%)	1
40–59	2392 (60.3%)	1609 (58.1%)	783 (65.3%)	1.01 (0.80–1.28)
≥60	1097 (27.7%)	818 (29.6%)	279 (23.3%)	1.68 (1.29–2.19) *
**Ethnicity**				
Chinese	2627 (66.2%)	1572 (56.8%)	1055 (88.0%)	1
Malay	826(20.8%)	750(27.1%)	76 (6.3%)	6.25 (4.85–8.06) *
Indian	481 (12.1%)	417 (15.1%)	64 (5.3%)	4.20 (3.17–5.56) *
Other	32 (0.8%)	28 (1.0%)	4 (0.3%)	4.80 (1.66–13.87) *
**Stage**				
Stage I	977 (24.6%)	598 (21.6%)	379 (31.6%)	1
Stage II	1555 (39.2%)	1040 (37.6%)	515 (43.0%)	1.20 (1.01–1.44) *
Stage III	993 (25.0%)	747 (27.0%)	246 (20.5%)	1.71 (1.39–2.11) *
Stage IV	434 (10.9%)	376 (13.6%)	58 (4.8%)	3.30 (2.38–4.58) *
Unknown	7 (0.2%)	6 (0.2%)	1 (0.1%)	1.45 (0.16–13.49)
**Tumor size (cm)**				
Median (range)	3 (0–31)	3 (0–31)	2.5 (0–20)	
<2	759 (19.1%)	457 (16.5%)	302 (25.2%)	1
2–5	2187 (55.1%)	1489 (53.8%)	698 (58.2%)	1.27 (1.05–1.53) *
>5	829 (20.9%)	691 (25.0%)	138 (11.5%)	2.19 (1.65–2.90) *
Unknown	191 (4.8%)	130 (4.7%)	61 (5.1%)	1.33 (0.92–1.91)
**Involved axillary nodes**				
0	1858 (46.8%)	1203 (43.5%)	655 (54.6%)	1
1–3	764 (19.3%)	514 (18.6%)	250 (20.9%)	1.02 (0.84–1.24)
4–9	402 (10.1%)	286 (10.3%)	116 (9.7%)	0.98 (0.75–1.27)
≥10	332 (8.4%)	250 (9.0%)	82 (6.8%)	1.18 (0.88–1.59)
Unknown	610 (15.4%)	514 (18.6%)	96 (0.8%)	1.48 (1.08–2.02) *
**Distant metastasis**				
No ^b^	3532 (89.1%)	2391 (86.4%)	1141 (95.2%)	1
Yes	434 (10.9%)	376 (13.6%)	58 (4.8%)	1.66 (1.19–2.32) *
**Grade**				
Good	289 (7.3%)	192 (6.9%)	97 (8.1%)	1
Moderate	1483 (37.4%)	987 (35.7%)	496 (41.4%)	0.94 (0.71–1.26)
Poor	1198 (30.2%)	844 (30.5%)	354 (29.5%)	1.04 (0.76–1.42)
Unknown	996 (25.1%)	744 (26.9%)	252 (21.0%)	1.03 (0.75–1.41)
**Estrogen receptor status**				
Negative	1474 (37.2%)	1048 (37.9%)	426 (35.5%)	1
Positive	2252 (56.8%)	1538 (55.6%)	714 (59.5%)	0.87 (0.70–1.08)
Unknown	240 (6.1%)	181 (6.5%)	59 (4.9%)	0.20 (0.11–0.39) *
**Progesterone receptor status**				
Negative	1671 (42.1%)	1159 (41.9%)	512 (42.7%)	1
Positive	1874 (47.3%)	1262 (45.6%)	612 (51.0%)	1.15 (0.93–1.42)
Unknown	421 (10.6%)	346 (12.5%)	75 (6.3%)	3.47 (2.09–5.75) *
**HER2 status**				
Negative	1933 (48.7%)	1341 (48.5%)	592 (49.4%)	1
Positive	1086 (27.4%)	768 (27.8%)	318 (26.5%)	0.97 (0.81–1.16)
Equivocal	513 (12.9%)	313 (11.3%)	200 (16.7%)	0.64 (0.51–0.80) *
Unknown	434 (10.9%)	345 (12.5%)	89 (7.4%)	1.19 (0.77–1.82)

Note. HER2 = Human Epidermal Growth Factor Receptor 2; ^a^ AOR = Adjusted odds ratio derived using logistic regression model with type of hospital as outcome (UMMC vs. UMSC). The model for stage was adjusted for age, ethnicity, tumor grade, estrogen receptor status, progesterone receptor status and HER2 status. All other models were additionally adjusted for tumor size, number of involved axillary nodes, and distant metastasis to account for stage; ^b^ Including patients with unknown status for distant metastasis (n = 5); * Statistically significant (*p*-value < 0.05). UMMC = University Malaya Medical Centre UMSC = University Malaya Specialist Centre. OR = Odds Ratio. CI = Confidence Interval.

**Table 2:** Distribution of treatment administered by hospital type.

	Overall n, %	UMMC n, %	UMSC n, %	Adjusted OR (95% CI) ^a^
**Surgery** ^b^				
Overall				
No	285 (8.1%)	233 (9.8%)	52 (4.6%)	
Yes	3240 (91.9%)	2152 (90.2%)	1088 (95.4%)	0.88 (0.43–1.79) ^c^
Type of surgery *				
Yes, mastectomy	2412 (74.4%)	1644 (76.4%)	768 (70.6%)	
Yes, breast conserving surgery	824 (25.4%)	508 (23.6%)	316 (29.0%)	
Yes, unknown	4 (0.1%)	0	4 (0.4%)	
**Radiotherapy** ^b^				
Overall				
No	1204 (37.9%)	850 (38.7%)	354 (36.2%)	
Yes	1970 (62.1%)	1347 (61.3%)	623 (63.8%)	0.83 (0.70–0.99) ^d^
Unknown	351	188	163	
By surgical status				
Yes, after breast conserving surgery *	694 (84.2%)	443 (87.2%)	251 (79.4%)	
Yes, after mastectomy *	1263 (52.4%)	893 (54.3%)	370 (48.2%)	
Yes, with no surgery	13 (4.6%)	11 (4.7%)	2 (3.8%)	
**Chemotherapy**				
Overall				
No	1421 (36.7%)	1003 (36.4%)	418 (37.4%)	
Yes	2451 (63.3%)	1751 (63.6%)	700 (62.6%)	1.18 (0.99–1.40) ^e^
Unknown	94	13	81	
By stage				
Yes, stage I–III *	2182 (61.9%)	1521 (63.8%)	661 (58.0%)	
Yes, stage IV	268 (61.8%)	229 (60.9%)	39 (67.2%)	
Yes, stage unknown	1 (12.5%)	1 (14.3%)	0	
Type of chemotherapy regime *^,f^				
1st generation (CMF)	55 (2.6%)	48 (3.1%)	7 (1.2%)	
2nd generation (anthracycline-based)	1616 (76.4%)	1295 (83.7%)	321 (56.6%)	
3rd generation (taxane-based)	444 (21.0%)	205 (13.2%)	239 (42.2%)	
Unknown	336	203	133	
**Hormone therapy**				
Overall				
No	1243 (36.0%)	919 (37.4%)	324 (32.7%)	
Yes	2208 (64.0%)	1540 (62.6%)	668 (67.3%)	0.90 (0.77–1.06) ^g^
Unknown	515	308	207	
By ER status				
Yes, ER positive *	1982 (88.0%)	1366 (88.8%)	616 (86.3%)	
Yes, ER negative *	176 (11.9%)	137 (13.1%)	39 (9.2%)	
Yes, ER status unknown	50	37 (20.4%)	13 (22.0%)	

* Statistically significant (*p* < 0.001); ^a^ Derived using a multivariable logistic regression model with the respective treatment as outcome (yes vs. no); ^b^ Excluding patients with stage IV breast cancer or unknown stage; ^c^ Includes 3525 patients with stage I to stage III breast cancer. Adjusted odds ratio of receiving surgery in UMMC to UMSC. Model was adjusted for ethnicity, tumor size (cm), number of involved axillary nodes, and estrogen and progesterone receptor status; ^d^ Includes 3174 patients with known adjuvant radiotherapy status. Adjusted odds ratio of receiving adjuvant radiotherapy in UMMC to UMSC. Model was adjusted for tumor size (cm) and number of involved axillary nodes; ^e^ Includes 3872 patients with known chemotherapy status. Adjusted odds ratio of receiving chemotherapy in UMMC to UMSC. Model was adjusted for age, tumor size (cm), number of involved axillary nodes and distant metastasis (yes vs. no); ^f^ Includes 2451 patients who received chemotherapy; ^g^ Includes 3451 patients with known hormone therapy status. Adjusted odds ratio of hormone therapy in UMMC to UMSC. Model was adjusted for tumor size (cm), number of involved axillary nodes and distant metastasis (yes vs. no). CMF = Cyclophosphamide, Methotrexate, 5-Fluorouracil, ER = Estrogen Receptor.

**Table 3:** Overall and stage-stratified survival estimates by hospitals.

	Five-Year Survival Estimate		Ten-Year Survival Estimate	
	**UMMC**	**UMSC**	**UMMC**	**UMSC**
**Overall ^a^**	71.6% (69.8%–73.4%)	86.8% (84.8%–88.8%)	52.3% (50.1%–54.5%)	76.6% (73.7%–79.5%)
Number of patients	2763	1193	2763	1193
Number of deaths	784	158	1144	233
**By Stage**				
Stage I ^b^	92.6% (90.4%–94.8%)	95.8% (93.8%–97.8%)	82.7% (79.2%–86.2%)	89.4% (85.7%–93.1%)
Number of patients	597	378	597	378
Number of deaths	44	16	82	31
Stage II ^a^	83.5% (81.1%–85.9%)	91.2% (88.8%–93.6%)	64.3% (61.0%–67.6%)	81.4% (77.5%–85.3%)
Number of patients	1038	513	1038	513
Number of deaths	171	45	306	78
Stage III ^a^	62.6% (59.1%–66.1%)	74.9% (69.4%–80.4%)	37.9% (34.0%–41.8%)	59.2% (51.6%–66.8%)
Number of patients	746	243	746	243
Number of deaths	279	61	411	83
Stage IV ^c^	23.4% (19.1%–27.7%)	37.9% (25.4%–50.4%)	2.5% (0.5%–4.5%)	14.6% (−1.1%–30.3%)
Number of patients	376	58	376	58
Number of deaths	288	36	343	41

^a^ Log rank test *p*-value < 0.001; ^b^ Log rank test *p*-value = 0.036; ^c^ Log rank test *p*-value = 0.005.

**Table 4:** Hazard ratio (HR) of all-cause mortality for invasive patients using stepwise Cox regression analysis.

	Total	UMSC	UMMC	Change in HR from Prior Model
Number of patients	3956	1193	2763	
Number of deaths	1436	240	1196	
Hazard ratio model A (95% CI) ^a^		1 (ref)	2.22 (1.94–2.55)	
Hazard ratio model B (95% CI) ^b^		1 (ref)	2.06 (1.78–2.38)	7.21%
Hazard ratio model C (95% CI) ^c^		1 (ref)	1.65 (1.43–1.91)	19.90%
Hazard ratio model D (95% CI) ^d^		1 (ref)	1.59 (1.36–1.85)	3.64%

^a^ Unadjusted hazard ratio; ^b^ Adjusted for age, ethnicity and year of diagnosis; ^c^ Adjusted for variables in model B plus tumor size (cm), number of regional nodes positive and distant metastasis (yes vs. no); ^d^ Adjusted for variables in model C plus locoregional treatment (no surgery, mastectomy only, mastectomy plus radiotherapy, breast conserving surgery only, breast conserving surgery plus radiotherapy), chemotherapy (no chemotherapy, 1st generation chemotherapy, 2nd generation chemotherapy, 3rd generation chemotherapy) and hormone therapy (no hormone therapy, hormone therapy).
